# Fetal Cardiovascular Decompensation During Labor Predicted From the Individual Heart Rate Tracing: A Machine Learning Approach in Near-Term Fetal Sheep Model

**DOI:** 10.3389/fped.2021.593889

**Published:** 2021-05-05

**Authors:** Nathan Gold, Christophe L. Herry, Xiaogang Wang, Martin G. Frasch

**Affiliations:** ^1^Department of Mathematics and Statistics, York University, Toronto, ON, Canada; ^2^Centre for Quantitative Analysis and Modelling, Fields Institute for Research in Mathematical Science, Toronto, ON, Canada; ^3^Dynamical Analysis Laboratory, Clinical Epidemiology Program, Ottawa Hospital Research Institute, Ottawa, ON, Canada; ^4^Institute of Big Data, Qing Hua University, Beijing, China; ^5^Department of Obstetrics and Gynecology and Center on Human Development and Disability, University of Washington, Seattle, WA, United States

**Keywords:** HRV, hypotension, brain Injury, Bezold Jarisch reflex, machine learning, time series, anomaly detection, changepoint detection

## Abstract

**Background:** When exposed to repetitive umbilical cord occlusions (UCO) with worsening acidemia, fetuses eventually develop cardiovascular decompensation manifesting as pathological hypotensive arterial blood pressure (ABP) responses to fetal heart rate (FHR) decelerations. Failure to maintain cardiac output during labor is a key event leading up to brain injury. We reported that the timing of the event when a fetus begins to exhibit this cardiovascular phenotype is highly individual and was impossible to predict.

**Objective:** We hypothesized that this phenotype would be reflected in the individual behavior of heart rate variability (HRV) as measured by root mean square of successive differences of R-R intervals (RMSSD), a measure of vagal modulation of HRV, which is known to increase with worsening acidemia. This is clinically relevant because HRV can be computed in real-time intrapartum. Consequently, we aimed to predict the individual timing of the event when a hypotensive ABP pattern would emerge in a fetus from a series of continuous RMSSD data.

**Study Design:** Fourteen near-term fetal sheep were chronically instrumented with vascular catheters to record fetal arterial blood pressure, umbilical cord occluder to mimic uterine contractions occurring during human labor and ECG electrodes to compute the ECG-derived HRV measure RMSSD. All animals were studied over a ~6 h period. After a 1–2 h baseline control period, the animals underwent mild, moderate, and severe series of repetitive UCO. We applied the recently developed machine learning algorithm to detect physiologically meaningful changes in RMSSD dynamics with worsening acidemia and hypotensive responses to FHR decelerations. To mimic clinical scenarios using an ultrasound-based 4 Hz FHR sampling rate, we recomputed RMSSD from FHR sampled at 4 Hz and compared the performance of our algorithm under both conditions (1,000 Hz vs. 4 Hz).

**Results:** The RMSSD values were highly non-stationary, with four different regimes and three regime changes, corresponding to a baseline period followed by mild, moderate, and severe UCO series. Each time series was characterized by seemingly randomly occurring (in terms of timing of the individual onset) increase in RMSSD values at different time points during the moderate UCO series and at the start of the severe UCO series. This event manifested as an increasing trend in RMSSD values, which counter-intuitively emerged as a period of relative stationarity for the time series. Our algorithm identified these change points as the individual time points of cardiovascular decompensation with 92% sensitivity, 86% accuracy and 92% precision which corresponded to 14 ± 21 min before the visual identification. In the 4 Hz RMSSD time series, the algorithm detected the event with 3 times earlier detection times than at 1,000 Hz, i.e., producing false positive alarms with 50% sensitivity, 21% accuracy, and 27% precision. We identified the overestimation of baseline FHR variability by RMSSD at a 4 Hz sampling rate to be the cause of this phenomenon.

**Conclusions:** The key finding is demonstration of FHR monitoring to detect fetal cardiovascular decompensation during labor. This validates the hypothesis that our HRV-based algorithm identifies individual time points of ABP responses to UCO with worsening acidemia by extracting change point information from the physiologically related fluctuations in the RMSSD signal. This performance depends on the acquisition accuracy of beat to beat fluctuations achieved in trans-abdominal ECG devices and fails at the sampling rate used clinically in ultrasound-based systems. This has implications for implementing such an approach in clinical practice.

A. **Why was the study conducted?**
  During labor, fetuses may develop pathologically hypotensive arterial blood pressure responses to fetal heart rate (FHR) decelerations triggered by uterine contractions. The timing of this event is difficult to predict clinically. We developed a machine learning method to detect this event from an individual FHR tracing.
B. **What are the key findings?**
  This real-time algorithm performs well on noisy FHR data requiring ~2 hours to train on the individual FHR tracings in the first stage of labor; once trained, the algorithm predicts the event with 92% sensitivity, 86% accuracy, and 92% precision.
  The algorithm's performance deteriorates to 50% sensitivity, 21% accuracy, and 27% precision when the FHR is acquired at a sampling rate of 4 Hz used in the ultrasound (CTG) monitors compared to the ECG-derived signal as it can be acquired from maternal abdominal ECG.
C. **What does this study add to what is already known?**
  This is the first demonstration of the ability to detect fetal cardiovascular decompensation, a prequel to brain injury, intrapartum. The approach is ready for clinical testing. Computerized CTG monitoring cannot predict fetal acidemia intrapartum as well as ECG-based FHR monitoring. This study adds to this knowledge that a computerized approach for objective detection of cardiovascular compromise from FHR in real-time from an individual FHR tracing also performs better when using ECG-derived FHR tracing than CTG tracing.

## Introduction

Electronic fetal monitoring (EFM) cannot identify fetuses at risk of incipient brain injury. The efforts to identify intrapartum acidemia using EFM have failed, in particular using fetal heart rate (FHR) monitoring, because fetal brain injury is poorly correlated with acidemia (1). Brain compromise due to hypoxia-ischemia (HI) can ensue when the fetal cerebral blood flow is persistently reduced e.g., due to precipitous drop in cerebral perfusion pressure resulting from cardiovascular decompensation (2, 3). Bezold-Jarisch reflex (BJR) is a vagal depressor reflex observed in fetal sheep under the conditions of umbilical cord occlusions (UCO) with worsening acidemia which leads to cardiovascular decompensation (4). We asked whether FHR monitoring can capture the BJR-mediated vagal sensing of acidemia. We studied the relationship between fetal systemic arterial blood pressure (ABP) and FHR in an animal model of human labor.

We had reported that sheep fetuses have an individual cardiovascular phenotype in their responses to increasing acidemia due to repetitive intermittent hypoxia (3). We hypothesized that such phenotype would be reflected in individual responses of heart rate variability (HRV) as measured by root mean square of successive differences of R-R intervals (RMSSD), a measure of vagal modulation of HRV known to increase with worsening acidemia (5–7). Consequently, a series of continuously computed RMSSD data will consistently predict the event when a hypotensive ABP pattern emerges in an individual fetus (3).

The current standard of EFM relies predominantly on ultrasound-based FHR monitoring. Because the vagally mediated HRV is found on a time scale that is not captured at 4 Hz sampling rate, we also tested the impact of its inherently lower FHR sampling rate precision of 4 Hz vs. the golden standard electrocardiogram (ECG)—derived 1,000 Hz on the ability to individually predict cardiovascular decompensation. We hypothesized that the lower temporal precision will result in a poorer prediction of the timing of cardiovascular decompensation.

## Materials and Methods

Experimental methods and data acquisition have been presented elsewhere (8). Briefly, fourteen near-term fetal sheep were chronically instrumented with vascular catheters to record fetal arterial blood pressure, umbilical cord occluder to mimic uterine contractions occurring during human labor and ECG electrodes to compute ECG-derived HRV measure RMSSD. Animal care followed the guidelines of the Canadian Council on Animal Care and was approved by the University of Western Ontario Council on Animal Care.

### Surgical Preparation

Fourteen near-term ovine fetuses [123 ± 2 days gestational age (GA), term = 145 days] of the mixed breed were surgically instrumented. The anesthetic and surgical procedures and postoperative care of the animals have been previously described (3, 9). Briefly, polyvinyl catheters were placed in the right and left brachiocephalic arteries, the cephalic vein, and the amniotic cavity. Stainless steel electrodes were sewn onto the fetal chest to monitor the electrocardiogram (ECG). A polyvinyl catheter was also placed in the maternal femoral vein. Stainless steel electrodes were additionally implanted biparietally on the dura for the recording of electrocorticogram, ECOG, as a measure of summated brain electrical activity [results reported elsewhere (3, 8, 10)]. An inflatable silicon rubber cuff (*In vivo* Metric, Healdsburg, CA) for UCO induction was placed around the proximal portion of the umbilical cord and secured to the abdominal skin. Once the fetus was returned to the uterus, a catheter was placed in the amniotic fluid cavity. Antibiotics were administered intravenously to the mother (0.2 g of trimethoprim and 1.2 g sulfadoxine, Schering Canada Inc., Pointe-Claire, Canada) and fetus and into the amniotic cavity (1 million IU penicillin G sodium, Pharmaceutical Partners of Canada, Richmond Hill, Canada). Amniotic fluid lost during surgery was replaced with warm saline. The uterus and abdominal wall incisions were sutured in layers and the catheters exteriorized through the maternal flank and secured to the back of the ewe in a plastic pouch.

Postoperatively, animals were allowed 4 days to recover prior to experimentation and daily antibiotic administration was continued intravenously to the mother (0.2 g trimethoprim and 1.2 g sulfadoxine), into the fetal vein and the amniotic cavity (1 million IU penicillin G sodium, respectively). Arterial blood was sampled for evaluation of the maternal and fetal condition and catheters were flushed with heparinized saline to maintain patency. Animals were 130 ± 1 day GA on the first day of the experimental study.

### Experimental Procedure

**Figure 1:**
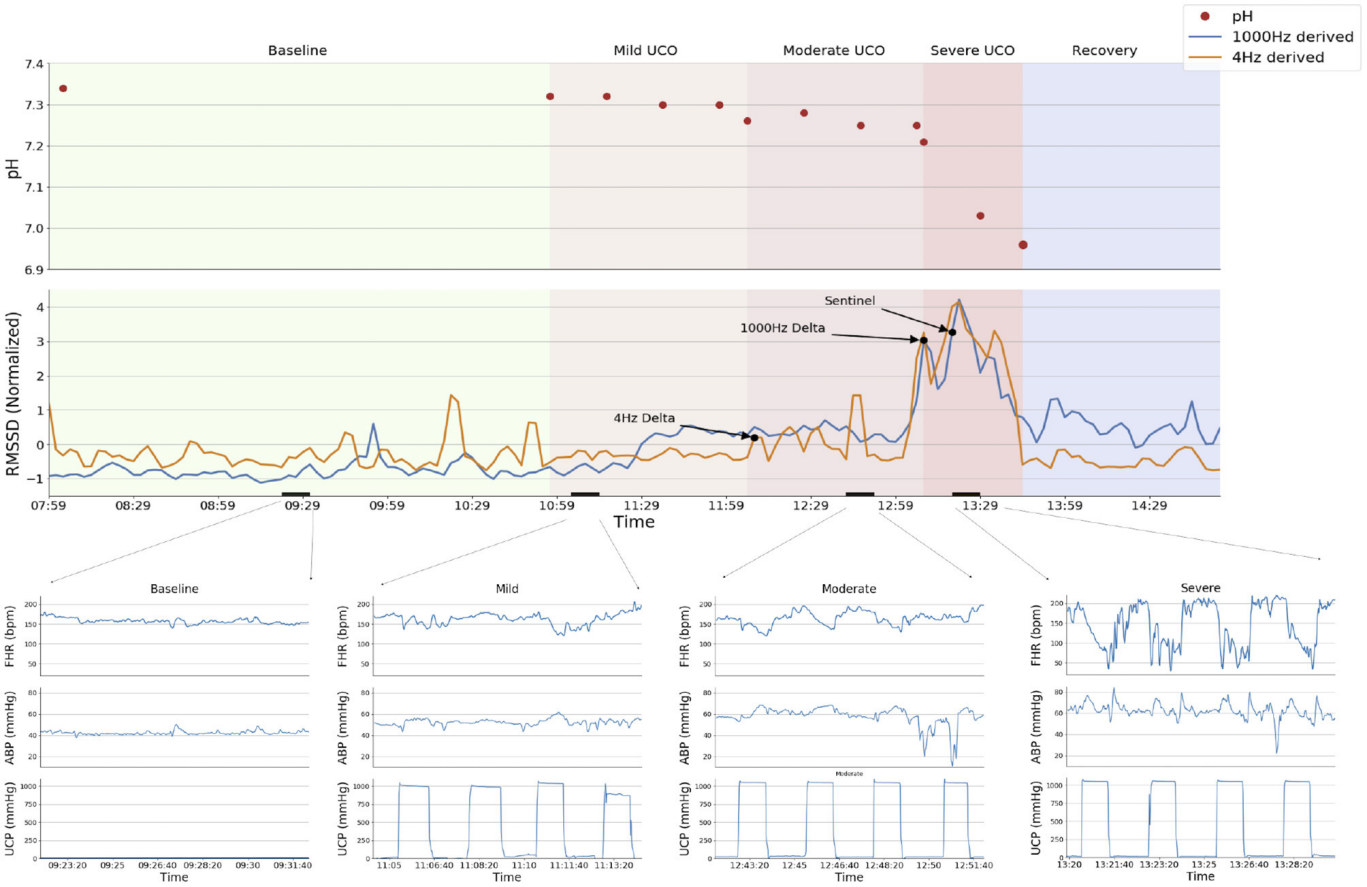
Experimental protocol and a representative example of the analytical approach (animal ID 473378). The RMSSD time series derived from 1,000 Hz (blue) and 4 Hz (orange) sampled ECG are displayed superimposed in the top panel, with declared change points from the BOCPD algorithm and the Sentinel (expert detection) marked with arrows. Sequential fetal arterial pH measurements are indicated. Experimental stages are demarcated by background colors; short black bars over X-axis indicate the zoomed-in segments shown in the bottom panel. Bottom panels show fetal heart rate (FHR, bpm), fetal arterial blood pressure (ABP, mmHg) and umbilical cord pressure (UCP, mmHg) indicating when UCO were triggered (increasing UCP). Note the failure of the change point algorithm to detect the sentinel time point correctly (i.e., around the Sentinel time point) when using 4 Hz—derived RMSSD signal: the detection occurs 1 h earlier than with the 1,000 Hz signal. This is due to unphysiological fluctuations in FHR variability at baseline as demonstrated in Durosier et al. (7) and Li et al. (19).

All animals were studied over a ~6 h period (Figure 1). Fetal chronic hypoxia was defined as arterial O_2_Sat <55% as measured on postoperative days 1–3 and at baseline prior to beginning the UCOs. The first group comprised five fetuses that were also spontaneously hypoxic (*n* = 5, H/UCO). The second group of fetuses was normoxic (O_2_Sat>55% before UCOs) (*n* = 9, N/UCO). The experimental protocol has been reported (7, 9, 11). After a 1–2 h baseline control period, the animals underwent mild, moderate, and severe series of repetitive UCOs by graduated inflation of the occluder cuff with a saline solution. During the first hour following the baseline period, mild variable FHR decelerations were performed with a partial UCO for 1 min duration every 2.5 min, with the goal of decreasing FHR by ~30 bpm, corresponding to a ~50% reduction in umbilical blood flow (12, 13). During the second hour, moderate variable FHR decelerations were performed with increased partial UCO for 1 min duration every 2.5 min with the goal of decreasing FHR by ~60 bpm, corresponding to a ~75% reduction in umbilical blood flow (13). Animals underwent severe variable FHR decelerations with complete UCO for 1 min duration every 2.5 min until the targeted fetal arterial pH of <7.0 was detected or 2 h of severe UCO had been carried out, at which point the repetitive UCOs were terminated. These animals were then allowed to recover for 48 h following the last UCO. Fetal arterial blood samples were drawn at baseline, at the end of the first UCO of each series (mild, moderate, severe), and at 20 min intervals (between UCOs) throughout each of the series, as well as at 1, 24, and 48 h of recovery. For each UCO series blood gas sample and the 24 h recovery sample of 0.7 ml of fetal blood was withdrawn, while 4 ml of fetal blood was withdrawn at baseline, at pH_nadir_ < 7.00, and at 1 and 48 h of recovery. The amounts of blood withdrawn were documented for each fetus and replaced with an equivalent volume of maternal blood at the end of day 1 of the study.

All blood samples were analyzed for blood gas values, pH, glucose, and lactate with an ABL-725 blood gas analyzer (Radiometer Medical, Copenhagen, Denmark) with temperature corrected to 39.0°C. Plasma from the 4 ml blood samples was frozen and stored for cytokine analysis, reported elsewhere.

After the 48 h recovery blood sample, the ewe and the fetus were killed by an overdose of barbiturate (30 mg sodium pentobarbital IV, MTC Pharmaceuticals, Cambridge, Canada). A post mortem was carried out during which fetal sex and weight were determined and the location and function of the umbilical occluder were confirmed. The fetal brain was perfusion-fixed and subsequently dissected and processed for later immunohistochemical study as reported (14).

### Data Acquisition and Analysis

A computerized data acquisition system was used to record fetal systemic arterial and amniotic pressures and the ECG signal, as described (7). All signals were monitored continuously throughout the experiment. Arterial and amniotic pressures were measured using Statham pressure transducers (P23 ID; Gould Inc., Oxnard, CA). Arterial blood pressure (ABP) was determined as the difference between instantaneous values of arterial and amniotic pressures. A PowerLab system was used for data acquisition and analysis (Chart 5 For Windows, ADInstruments Pty Ltd, Castile Hill, Australia). Pressures, ECOG and ECG were recorded and digitized at 1,000 Hz for further study. For ECG, a 60 Hz notch filter was applied.

R peaks of ECG were used to derive the heart rate variability (HRV) times series. Beat detection was performed using a mix of two algorithms, a custom wavelet-based detection and Elgendi's method with an added refractory period step (15). Both methods include bandpass filtering as an initial step that removes baseline wandering and high frequency noise. Beat detection was also verified for accuracy using a custom developed ECG annotation and reviewing tool. This was necessary to validate beat detection in UCO periods where the noise level was high and where there were artifacts generated by the contractions. R-R intervals were further filtered based on the morphology of the ECG waveforms, the level of noise/artifacts within short windows and the proportion of disconnected/saturated periods, if any (16, 17). Windows of low quality were not retained in the HRV analysis. Low quality was defined over analysis windows (5 min) as a weighted sum of the percentage of time without non-physiologic beats (artifacts), the percentage of time uninterrupted by disconnections/saturations, the percentage of time with high quality beats according to Clifford et al.'s (16, 17). Ectopies were not filtered out as there were a large number of them during UCO periods and it would effectively remove most of the UCO periods if filtered out. The average percentage of original ECG signal ultimately discarded for the HRV analysis was 5.5% (range: 1–20%).

The time series of R-R peak intervals were uniformly resampled at 4 Hz. Technically, the resampling was performed as an interpolation since we need to go from a pseudo-frequency of 2.5–3 Hz to a sampling frequency of 4 Hz. The interpolation method used was a piecewise cubic Hermite interpolation. Next, the RMSSD was calculated continuously on both the original R-R interval time series (with 1-millisecond resolution) and the R-R interval time series resampled at 4 Hz, from each 5 min HRV segment in 2.5 min overlapping sliding windows. For the ~6-h time series, this corresponded to roughly 150 data points.

During UCO series, the point at which hypotensive ABP responses to UCO had been detected by “expert” visual detection was termed ABP “sentinel,” defined as the time between the onset of such ABP responses to UCO and the time when pH nadir (pH < 7.00) is reached in each fetus.

To detect changes in RMSSD values corresponding to the above sentinel time point in the ABP responses, we used the previously reported machine learning algorithm, referred to as Delta point method, based on change point detection (18). Briefly, Delta point method is a real-time change point detection method, robust to false-alarms, designed to filter a vector of suspected change points. It proceeds by fitting a probabilistic Gaussian process model to the RMSSD time series baseline data and computing online predictions of the RMSSD values within the range of the model. Suspected change-points are declared as significant (*p* < 0.05) deviations from pointwise model predictions and observations. These are viewed as observations of a doubly stochastic Poisson process, with observation rate governed by the Gaussian process model. Based upon this theory, the points are grouped into time intervals, within which the Delta point is selected as the most significant change.

To perform hyper-parameter training, we segmented the data into a 60 point training set, or 2.5-h training time on the baseline and mild UCO periods of each time series (i.e., corresponding to the first stage of labor). Our method uses an n = 10 point or 25-min interval to segment the time series for delta point evaluation. The choice of 10 points or 25-min interval is to provide a reasonable number of points per interval for the Delta point method, so that a reasonable average may be calculated for the average run in each interval.

We defined a successful detection as the agreement between the Delta point and the sentinel value, with Delta point detection no later than 2 min behind expert detection. False negative detections were defined as Delta point being declared 2 min behind expert detection. False positive detections were defined as detection occuring 25 min prior to expert detection, corresponding to one Delta point sampling interval. This demonstrates the effectiveness of the method, suggesting clinical benefits for earlier decision making.

### Statistical Analysis

The differences in the change point detection at 4 Hz compared to 1,000 Hz were evaluated with the Wilcoxon signed-rank test with a *P* < 0.05 was considered significant. Detection performance was analyzed by computing the accuracy, sensitivity, and precision of the method defined as,

(1) $$\text{Accuracy }=\frac{\text{Successful detections}}{\text{Number of examples}}$$

(2) $$Sensitivity\;=\;\frac{True\;Positive}{True\;Positive\;+\;False\;Negative}$$

(3) $$Precision\;=\;\frac{True\;Positive}{True\;Positive\;+\;False\;Positive}$$

### Results

The physiological characteristics of the experimental groups have been reported (8, 10, 11).

Delta point method was able to match the expert prediction with Delta point declaration occurring at a median 8.5 (IQR = 10.5) minutes before ABP sentinel time. This corresponded to 92% sensitivity, 86% accuracy, and 92% precision.

In the 4 Hz RMSSD time series, the algorithm triggered change point at a median 36 (IQR = 44.3) minutes failing to match the expert prediction by yielding 8 times earlier detection times than at 1,000 Hz, i.e., producing false positive alarms in 8 out of 14 cases (*p* = 0.003). This corresponded to 50% sensitivity, 21% accuracy, and 27% precision. We report the confusion matrix for both the 1,000 Hz RMSSD and 4 HZ RMSSD time series in Table 1.

**Table 1:** Confusion matrix.

**1,000 Hz**	**Positive**	**Negative**	**4 Hz**	**Positive**	**Negative**
**Positive**	12	1	**Positive**	3	8
**Negative**	1	0	**Negative**	3	0

A representative example of the experimental data is shown in Figure 1 and the individual findings for all subjects are reported in Table 2.

**Table 2:** Performance of the anomaly detection algorithm in predicting the individual time points of cardiovascular decompensation from FHR.

**Group**	**Animal**	**Sentinel**	**1,000 Hz detection**	**4 Hz detection**	**1,000 Hz delta**	**4 Hz delta**
H_UCO	8003	15:56:00	15:49:00	15:52:00	0:07	0:04
H_UCO	473351	13:38:00	13:28:00	13:04:00	0:10	0:34
H_UCO	473362	11:05:00	11:03:00	11:35:00	0:02	0:30
H_UCO	473376	12:36:00	12:38:00	11:59:00	0:02	0:37
H_UCO	473726	12:04:00	11:50:00	11:54:00	0:14	0:10
N_UCO	461060	12:42:00	12:31:00	12:21:00	0:11	0:21
N_UCO	473361	12:51:00	12:36:00	12:16:00	0:15	0:35
N_UCO	473352	13:17:00	12:53:00	12:06:00	0:24	1:11
N_UCO	473377	12:12:00	12:14:00	12:50:00	0:02	0:38
N_UCO	473378	13:22:00	13:09:00	12:09:00	0:13	1:13
N_UCO	473727	11:03:00	11:10:00	11:08:00	0:07	0:05
N_UCO	5054	12:53:00	11:27:00	11:19:00	1:26	1:34
N_UCO	5060	11:26:00	11:24:00	10:29:00	0:02	0:57
N_UCO	473360	13:59:00	13:52:00	11:55:00	0:07	2:04

*Sentinel, time of detecting the onset of pathological ABP decreases during UCOs by an expert (visual analysis); 1,000 and 4 Hz detection, times of detecting the same using the change point algorithm on RMSSD data derived from 1,000 or 4 Hz sampled ECG; 1,000 delta and 4 Hz delta, the time difference (sentinel-1,000 or sentinel-4 Hz) between expert and change point algorithm detection performance: detection by the algorithm preceded in most cases the expert detection, median 8.5 (IQR = 10.5) minutes and median 36 (IQR = 44.3) minutes, respectively; Red font, cases when the algorithm detection happened after the expert detection; note that in the case of 4 Hz delta, 2 out of 3 instances the detection was more than 30min too late compared to ~3min too late in the three cases at 1,000 Hz*.

The visual inspection of the RMSSD tracings suggested that the overestimation of the baseline FHR variability by RMSSD at the 4 Hz sampling rate is the cause of this false detection phenomenon. To verify this assumption we determined the RMSSD values computed from the 1,000 Hz and 4 Hz sampled FHR data sets at baseline and during the UCO series. Confirming our hypothesis, we found a smaller difference in the average normalized RMSSD values during the UCO series compared to the baseline in the 4 Hz data set (0.52 ± 0.16) compared to the 1,000 Hz data set (0.85 ± 0.4, *p* = 0.027).

## Discussion

### Principal Findings

Our findings validate the hypothesis that Delta point method, applied to the FHR-derived HRV measure RMSSD, identifies individual time points of ABP responses to UCO with worsening acidemia by extracting change point information from the physiologically related fluctuations in RMSSD time series. The present findings also show the dependence of this method on high temporal precision of FHR acquisition to capture correctly the physiological fluctuations of FHR at baseline. This is in line with the previous observations in the pregnant sheep model and human fetuses intrapartum (7, 19).

## Results

We had reported consistent changes in fetal brain electrical activity, the electrocorticogram (ECOG), with amplitude suppression and frequency increase during FHR decelerations accompanied by highly correlated pathological decreases in fetal ABP, referred to as adaptive brain shutdown (3). These changes in ECOG occurred on average 50 min prior to attaining a severe degree of acidemia (i.e., fetal arterial pH <7.00). However, we noted a high degree of inter-individual variability in the timing of the onset of these brain electrical and cardiovascular responses. Importantly for the neonatal outcome, we found a relationship between the ensuing neuroinflammation measured by the number of microglia, the brain's immune cells, and the timing of the adaptive brain shutdown onset (14). An individualized and timely detection of the onset of hypotensive responses to worsening acidemia and hence the timing of the adaptive brain shutdown would provide clinically relevant information on the degree of neuroinflammation after birth. Perinatal neuroinflammation has been identified as relevant prognostically not only short-term during early life, but also long-term for adult neurodevelopmental sequelae (20–29).

We suggest that the robust performance of the algorithm is owed to selecting causally linked phenomena which are reflected in the two different time series: RMSSD is known to rise with worsening acidemia due to chemoreceptors activation for example (6, 7). Meanwhile, fetal ABP responses to worsening acidemia deteriorate over time with an initial phase of hypertensive responses during each UCO to compensate for the drop in FHR, followed by the gradual decline of this hypertensive component and eventually ensuing pathological hypotensive ABP responses (3). This is at least partially due to a cardiac decompensation with growing levels of acidemia (30, 31). Acidemia impacts myocardial contractility which decreases cardiac output and ABP. It is plausible to expect that such transition in cardiac behavior will be reflected in HRV, RMSSD in particular, because HRV reflects not only the influences of the autonomic nervous system's vagal modulation of the cardiac sinus node activity, it also depends on the intrinsic cardiac rhythm fluctuations and health as evidenced by a decrease in HRV in patients after heart transplants and by presence of intrinsic HRV as early as in term fetuses of gestational age similar to the present study (31–35).

The RMSSD time series were highly non-stationary, with four different regimes and three regime changes, corresponding to a baseline period followed by mild, moderate and severe UCO series. Each time series was characterized by seemingly randomly occurring (in terms of timing of the individual onset) increase in RMSSD values at different time points during the moderate UCO series and at the start of the severe UCO series. This event manifested as an increasing trend in RMSSD values, which counter-intuitively emerged as a period of relative stationarity for the time series. The Delta point algorithm effectively declared these points as the change point of clinical importance. Overall, we found the Delta point algorithm's predictions to be reliable even in the instances when the signals were noisy (18). This is based on the tests of the algorithm in various data sets as published (18) and on our observation that here, to mimic the online recording situation, no correction for ectopies or non-sinus rhythms was undertaken on RMSSD as is usually done for HRV offline processing (34). To our knowledge, no comparable statistical or machine learning methods for FHR analysis exist.

The reliance on a high-quality RMSSD signal (i.e., derived from 1,000 Hz sampled true beat-to-beat variability signal) is also what explains the failure of the algorithm to detect relevant changepoints at 4 Hz sampling rate when the RMSSD signal becomes distorted due to undersampling and the resulting overestimation of baseline variability (7, 19).

### Clinical Implications

We demonstrate that computerized FHR monitoring intrapartum deploying machine learning can detect fetal cardiovascular decompensation during labor. Considering the average duration of labor of 12 h for nullipara, the requirement of a 2-h training window on the individual patient's data for the proposed algorithm is trivial (36, 37). Possible decision support such an algorithm can provide is alerting the healthcare provider to ease on contractions or to expedite the delivery to prevent fetal brain injury. Development of the actual clinical workflow will require retrospective and prospective clinical studies.

Our findings have direct clinical implications since high precision HRV can be recorded non-invasively in human fetuses from maternal abdominal ECG (38–42). Moreover, the present results validate and extend the insight we and others reported earlier in sheep and human fetuses whereby the reduced sampling rate of FHR acquisition decreases the precision of HRV—derived measures such as RMSSD for the detection of acidemia (7, 19, 43). Here, we show that the Delta point method performs 3-times more precisely in alerting to fetal cardiovascular decompensation when the underlying FHR signal was sampled at the gold standard 1,000 Hz rate available with today's fetal ECG monitors rather than at the 4 Hz rate as acquired with the ultrasound monitors.

### Research Implications

Future prospective clinical studies will investigate the utility of this discovery in the early detection of fetal cardiovascular compromise intrapartum using EFM. Our findings indicate the superiority of abdominal ECG-derived FHR signal for the prediction of cardiovascular decompensation. The present machine learning approach relies on the individual tracing to learn its properties and detect the timing of fetal cardiovascular compromise. That is, unlike most of the artificial intelligence (AI) technologies based on other machine learning methodologies or deep learning (artificial neural networks), our algorithm does not require a large amount of data from multiple subjects (thousands of subjects) to be fed into it in order to perform. Nevertheless, the advent of deep learning may also open new applications for more precise, individualized decision support using the conventional ultrasound-derived FHR tracings. In this context, future studies could focus on building big datasets of FHR recordings intrapartum to enable large scale testing of the AI-based algorithms such as the one presented here or the ones based on deep learning approaches, e.g., as recently pioneered in EFM by Georgieva et al. (44).

### Strengths and Limitations

The present findings from an established preclinical translational experiment present a conceptual advance in the clinical EFM demonstrating a novel machine learning approach for individualized detection of fetal cardiovascular compromise using FHR. The individual machine learning time of ~2 h during the first stage of labor is clinically realistic. The main limitation of the present study is that its insights are derived from an animal model paradigm, albeit well validated. As such, prospective human clinical studies of FHR intrapartum are needed. Such clinical studies will also shed light on our *a priori* choice of 25 min prior to the sentinel event as a cut-off for true positive detection. It is possible that an earlier detection and decision support for intervention in labor will be found beneficial for mother's and child's health. In such case, the 4 Hz-based conventional ultrasound FHR monitoring may turn out to also be amenable to such an algorithm. The risk of increasing the already alarmingly high rate of unnecessary cesarean sections speaks against this notion at this time. Furthermore, our approach so far took no advantage of the information contained in the changes in the uterine pressure during contractions in the first and second stages of pushing and the FHR response to it as is routinely done clinically during the FHR assessment. A combination of the present machine learning approach with information from uterine contractions will likely boost the performance of the presented algorithm in a clinical setting.

### Conclusions

The novelty of the current work is that its EFM algorithm permits statistical-level predictions about concomitant changes in individual FHR tracings which alert about fetal cardiovascular decompensation, an important mechanistic prequel to brain injury. The presented approach now awaits direct clinical validation in retrospective or prospective clinical studies.

## Condensation

Fetal heart rate (FHR) algorithm based on machine learning from individual FHR tracings detects early cardiovascular compromise in a sheep model of human labor.

## Data Availability Statement

The original contributions generated for the study are included in the article/Supplementary Material, further inquiries can be directed to the corresponding authors.

## Ethics Statement

The animal study was reviewed and approved by University of Western Ontario Council on Animal Care.

## Author Contributions

NG, XW, and MF conceived of the manuscript. NG, CH, and MF wrote the initial draft. NG and CH analyzed the data. NG, CH, XW, and MF contributed to the draft, read and approved the final version of the manuscript. MF holds patents on fetal EEG and ECG monitoring: US9,215,999 and WO2018160890. The authors have declared that no further conflict of interest exists.

## Conflict of Interest

CH is a co-inventor on patents related to physiological waveform assessment and variability analysis. MF holds patents on fetal EEG and ECG monitoring: US9,215,999 and WO2018160890. The remaining authors declare that the research was conducted in the absence of any commercial or financial relationships that could be construed as a potential conflict of interest.

## Supplementary Methods - Explanation of Delta Point Method

The Delta point method is a change point detection algorithm for the online interrogation of suspected change points in non-stationary time series. The algorithm proceeds by identifying suspected change points in a non-stationary time series by fitting a non-parametric function representation to the real-time observed time series data. The fitted functional representation then forecasts future values of the time series and a statistical algorithm is applied to the realized and forecasted values to determine if a significant divergence occurs between the respective values. When a statistically significant difference is observed, a suspected change point is declared.

Due to the noisy nature of biologically collected non-stationary time series, this noise often causes many false-positive detected change points for which real-time processing must be applied to determine the temporal location of a true change. The Delta point method uses the expanding returned vector of suspected change points and applies a windowing procedure to the temporal locations of the suspected changes. The rate of suspected change points is then fit to a doubly-stochastic point process to determine the rate at which suspected change points occur in the time series overall, as well as within each window. Due to the functional representation of the time series, this additional information is used to tune the estimated hazard rate of the point process. Based upon this representation, the window of interest is determined as the location with the greatest temporal difference between suspected change points. The Delta point - the change point of true interest - is then selected as the last occurring change point in the identified window.
